# Differentiation between Takotsubo syndrome and coronary spastic angina in subjects undergoing catheter ablation for atrial fibrillation

**DOI:** 10.1002/joa3.12908

**Published:** 2023-07-31

**Authors:** Hiroki Uehara, Takahiro Gunji

**Affiliations:** ^1^ Department of Cardiovascular Medicine Kin‐ikyo Chuo Hospital Sapporo Hokkaido Japan

## CONFLICT OF INTEREST STATEMENT

Authors declare no conflict of interests for this article.

I read with great interest the paper by Sinha et al. about Takotsubo syndrome (TS) in subjects undergoing catheter ablation for atrial fibrillation (AF) based on multicenter retrospective database.^1^ The author analyzed 69,116 subjects and stated that 27 subjects (0.04%) had a TS diagnostic code. The cohort study was compromised mostly of female [17 (63%)], and one (3.7%) death within 30 days was reported. TS in AF ablation did not occur as often as expected as the authors suggested, but I consider TS an important complication.

I have two comments and questions for the author: (1) Have you taken into consideration coronary spastic angina (CSA)? It is well known that CSA can be caused by direct thermal damage resulting from radiofrequency (RF) energy or an autonomic nervous system imbalance.^2^ Furthermore, perioperative sedation with dexmedetomidine can induce CSA.^3^ The authors' analysis lacked a summary of vasodilators like nitrates or the methods of sedation. CSA, particularly with a “wrap around” coronary artery, is frequently reported to mimic TS.^4^ MRI is regarded as a valuable tool for distinguishing between the two conditions. I suspect that the 27 subjects diagnosed with TS might include cases that should actually be diagnosed as CSA. If feasible, it would be reasonable to incorporate information about sedation methods and vasodilators, such as nitrates, in the analysis. (2) Do you consider 27 subjects diagnosed with TS to be a substantial finding? In a sizable retrospective study involving non‐cardiac surgery, encompassing 1460 liver transplant recipients, 17 patients (1.16%) developed postoperative cardiomyopathy.^5^ These patients did not exhibit myocardial ischemia and experienced reversible cardiomyopathy, strongly indicating TS. I posit that the emotional stress associated with hospitalization and the physical stress during the perioperative period can precipitate TS. Consequently, I believe there is no correlation between catheter ablation for AF and TS.
